# DAD3 targets ACE2 to inhibit the MAPK and NF-κB signalling pathways and protect against LPS-induced inflammation in bovine mammary epithelial cells

**DOI:** 10.1186/s13567-022-01122-0

**Published:** 2022-12-08

**Authors:** Xiangjun Zhang, Fang Jia, Weiwu Ma, Xueqiang Li, Xuezhang Zhou

**Affiliations:** 1 Key Laboratory of the Ministry of Education for the Conservation and Utilization of Special Biological Resources of Western China, Ningxia University, Yinchuan, 750021 Ningxia China; 2 Inner Mongolia Key Laboratory of Molecular Biology, School of Basic Medical Sciences, Inner Mongolia Medical University, Hohhot, 010110 China; 3 Key Laboratory of Energy Sources and Chemical Engineering, Development Center of Natural Products and Medication and School of Chemistry and Chemical Engineering, Ningxia University, Yinchuan, 750021 China

**Keywords:** Diminazene aceturate, diminazene aceturate derivative, bovine mammary epithelial cells, angiotensin-converting enzyme 2, inflammation

## Abstract

The protective arm of the renin-angiotensin system (RAS), the ACE 2/Ang-(1–7)/MasR axis, has become a new anti-inflammatory target. As a specific activator of ACE2, diminazene aceturate (DA) can promote anti-inflammatory effects by regulating the ACE2/Ang-(1–7)/MasR axis. However, due to the reported toxicity of DA, its application has been limited. In the current study, we synthesized a low toxicity DA derivative 3 (DAD3) and sought to determine whether DAD3 can also activate ACE2 in bovine mammary epithelial cells (BMEC) and regulate the RAS system to inhibit inflammation. We found that both DA and DAD3 can activate and promote ACE2 expression in BMEC. iRNA-mediated knockdown of ACE2 demonstrated that DAD3 activates the ACE2/Ang-(1–7)/MasR axis and plays an anti-inflammatory role in BMEC. Furthermore, the inhibitory effects of DA and DAD3 on the protein phosphorylation of MAPK and NF-κB pathways were reduced in ACE2-silenced BMEC. Our findings show that ACE2 is a target of DAD3, which leads to inhibition of the MAPK and NF-κB signalling pathways and protects against LPS-induced inflammation in BMEC. Thus, DAD3 may provide a new strategy to treat dairy cow mastitis.

**Supplementary Information:**

The online version contains supplementary material available at 10.1186/s13567-022-01122-0.

## Introduction

The renin-angiotensin system (RAS) is an important body fluid regulation system, that is predominantly distributed in blood vessel walls, heart, kidney, liver and eyes [1]. The main components of the RAS include angiotensinogen, angiotensin I (Ang I), Ang II, angiotensin converting enzyme (ACE), ACE2, angiotensin II type 1 receptor (AT1R), AT2R, Ang1-7, Ang1-9, and the Mas receptor (MasR). The system plays an important role in the regulation of cardiovascular function, the maintenance of body fluids and electrolyte balance, cell growth, tissue fibrosis, and regulation of the inflammatory immune response [2–4]. An imbalance in the secretion of RAS components can lead to the development of diseases associated with heart, kidneys and lungs [5–7], as well as inflammation. ACE2 plays a vital role in regulating the balance between the ACE2/Ang-(1–7)/MasR axis and ACE/Ang II/AT1R axis in RAS, in which the ACE/Ang II/AT1R axis exerts a pro-inflammatory effect, and the ACE2/Ang-(1–7)/MasR axis exerts an anti-inflammatory effect [8–11]. Studies have found that Ang II binds to AT1R, thereby activating the expression of downstream pro-inflammatory genes and the inflammatory response [8]. ACE2 can inhibit activation of the Ang II-dependent MAPK signalling pathway, and inhibit production of inflammatory factors, thereby playing an anti-inflammatory role in renal tubular epithelial cells [12]. Recombinant ACE2 can inhibit the production of inflammatory factors by regulating the JNK and NF-κB pathways, and alleviate the lipopolysaccharide (LPS)-induced inflammatory damage in pulmonary microvascular endothelial cells [13].

In addition to its anti-parasitic activity, diminazene aceturate (DA) has attracted much attention for its anti-inflammatory effect, which is associated with its ability to downregulate signalling pathways including MAPK and STAT that promote proinflammatory cytokine production [14]. Treatment of LPS-activated macrophages with DA, for example, led to a reduction in the phosphorylation levels of MAPK and STAT pathway proteins, decreased expression of pro-inflammatory cytokines, and reduced inflammatory damage [15]. Furthermore, as a specific activator of ACE2, DA promoted anti-inflammatory effects through regulation of the ACE2/Ang-(1–7)/MasR axis [16, 17]. Previous studies have shown that DA inhibits LPS-induced inflammatory injury of human retinal pigment epithelia (hRPE) by activating the ACE2/Ang-(1–7)/MasR axis and inhibiting the MAPK and NF-κB signalling pathways [18]. However, DA has also been reported to have toxic side effects [19]. For example, DA doses exceeding 10 mg/kg b.w. were found to cause severe damage in the gastrointestinal tract, respiratory, musculoskeletal, and nervous systems in camels and dogs [20]. In laboratory rodents, hypotension was also frequently observed, and gross and histopathological lesions have been described in the liver, kidneys, urinary bladder, lungs, heart and brain of dogs and camels at necropsy [21]. Reports regarding the toxic side effects of DA have led to the synthesis of a low-toxic DA derivative (DAD3), which has been shown to have strong anti-LPS-induced inflammatory effects on bovine mammary epithelial cells (BMEC), mediated predominantly through inhibition of the NF-κB and MAPK signalling pathways in previous study [22].

BMEC are the first line of defence against invasive bacteria in the mammary gland. Not only do they act as physical barriers, they are also capable of producing inflammatory mediators including cytokines when interacting with invasive bacteria [23]. However, excessive inflammation in the mammary gland can lead to tissue damage and clinical bovine mastitis [24]. Therefore, anti-inflammatories may be useful for the treatment of bovine mastitis.

In the current study, we sought to determine the role of ACE2 in mediating the DAD3-induced anti-inflammatory response, and further determine whether this response is mediated through inhibition of the NF-κB and MAPK signalling pathways. The aim is to provide new therapeutic strategies for the treatment of dairy cow mastitis.

## Materials and methods

### Cells and reagents

A BMEC-immortalized cell line was obtained from the College of Life Science, Shandong Agricultural University, China. BMEC were cultured in DMEM (DMEM/F-12 K, Hyclone, Logan, UT, USA) supplemented with 10% (vol/vol) foetal bovine serum (FBS, 16,000–44, Gibco, US origin). Ang II (MM-5103401) and Ang1-7 (MM-5103801) ELISA kits were purchased from Jiangsu Enzyme Industry Co., Ltd. (China). Anti-p38 (ab31828/Monoclonal/Mouse/IgG1/M138) was from Abcam (USA). Anti-phospho-p38 (MA5-15,182/Monoclonal/Rabbit/IgG/S.417.1) was from Thermos Fisher Scientific, Rockford (USA). Anti-IκB-α (9242S/Polyclonal/Rabbit) was purchased from CST (USA). Anti-phospho-IκB-α (bs-2513R/Polyclonal/Rabbit), anti-ERK(bs-0022R/Polyclonal/Rabbit), anti-phospho-ERK(bs-1522R/Polyclonal/Rabbit), anti-JNK(bs-2592R/Polyclonal/Rabbit), anti-phospho-JNK (bs-1640R/Polyclonal/Rabbit), anti-ACE2 (bs-23444R/Polyclonal/Rabbit) and anti-β-actin (bs-0061R/Polyclonal/Rabbit) were from Bioss (Beijing, China).

### Compound synthesis and cells treatment

Diminazene aceturate (DA, C_14_H_15_N_7_·2C_4_H_7_NO_3_, ab143391) was used as the mother nucleus, was purchased from Abcam (USA). DA derivative-DAD3 was synthesized as described previously [22]. Briefly, DA (281 mg), 2-methylbenzenesulfonyl chloride (190 mg) and acetonitrile (40 mL) were allowed to react at 80 °C for 3 days. After the reaction was completed, suction filtration was performed, the solid was rinsed with ethyl acetate and DAD3 was obtained (350 mg, 81.4% yield) (Additional file 1) ^1^H NMR (400 MHz, DMSO-d_6_) δ 13.24 (s, 1H), 9.33 (s, 4H), 8.99 (s, 4H), 7.90 (d,J = 8.3 Hz, 4H), 7.81–7.52 (m,4H), 7.24–7.03 (m, 3H), 2.50 (dd, J = 4.0, 2.2 Hz, 3H). ^13^ C NMR (101 MH_Z_, DMSO-d_6_) 165.23, 136.00, 131.20, 130.34, 129.21, 126.95, 125.30, 39.68, 20.61.

BMEC were cultured to the logarithmic growth phase, then seeded in 6-well plates (5 × 10^5^ cells/well) and grown for 24 h. Previously, the CCK-8 method has been used to determine non-cytotoxic concentrations of LPS, DA and DAD3 in BMEC [22]. Based on these concentrations, cells were treated with either LPS (0.5 μg/mL), DA (20 μg/mL), DAD3 (20 μg/mL), LPS+DA (0.5 μg/mL+20 μg/mL) or LPS+DAD3 (0.5 μg/mL+20 μg/mL) and incubated in serum-free culture medium for 24 h. Cells and culture supernatants were collected for ELISA, RT-qPCR and Western blot analysis.

### Localization of ACE2 in BMEC

BMEC were cultured to the logarithmic growth phase in 6-well plates (1 × 10^5^ cells/well) and incubated for 12 h. Samples were then fixed in 4% formaldehyde for 15 min and permeated with 0.5% TritonX-100 for 10 min. Next, samples were blocked with 5% BSA for 1 h, incubated with anti-ACE2 primary antibody (1:500), followed by CY3-labeled fluorescent secondary antibody (1:1000, bs-0295G-Cy3, Goat anti-rabbit, Polyclonal, Bioss, Beijing, China) for 2 h. Finally, samples were washed three times with PBS followed by incubation with DAPI (100 ng/mL) for 5 min in the dark. Images were observed under a laser confocal microscope (Leica Microsystems CMS GmbH, Enst-Leitz-Str. 17–37, D-35578 Wetzlar, Model: TL LED, Analysis software: LAS X).

### ACE2-siRNA transfection

ACE2 siRNA and negative control siRNA (scrambled siRNA) were synthesized by Shanghai Jima Pharmaceutical Technology Co., Ltd (China), and their gene sequences are shown in Table 1. siRNA transfection was carried out as described previously by Qiu et al. [25]. Briefly, BMEC were seeded in 6-well plates (2 × 10^5^ cells/well) overnight. siRNA was mixed with the Zeta Life transfection reagent (AD600150; USA) at a ratio of 1:1, and added to the cells for 24 h. Next, serum-free DMEM high glucose culture medium containing 0.5 μg/mL LPS was added to the cells for 24 h. Cells were washed with PBS, then treated with 20 μg/mL DA or 20 μg/mL DAD3 for 24 h. Samples were collected for subsequent analysis.

**Table 1:** **Sequences of small-interfering RNAs**

Gene	sense	antisense
ACE2-siRNA-155 ACE2-siRNA-2321	GGAGAAGUUUAACCAUGAATT GGUAGUGAUUGGUAUUGUUTT	UUCAUGGUUAAACUUCUCCTT AACAAUACCAAUCACUACCTT
Scrambled siRNA	UUCUCCGAACGUGUCACGUTT	ACGUGACACGUUCGGAGAATT

### RT-qPCR and ELISA

Total RNA was extracted from BMEC using Trizol reagent (TaKaRa-9108) according to the manufacturer’s instructions. cDNA was synthesized by reverse transcription using the PrimeScript™ RT reagent Kit with gDNA Eraser (TaKaRa-RR047A). The SYBR Premix Ex Taq™ II reagent (TaKaRa-RR820A) was used to perform the real-time (RT)-qPCR reaction. The primers used to amplify IL-1β, IL-6, IL-8, TNF-α and β-actin have been described previously [26, 27]. The primers used to amplify ACE2, MasR, AT1R and AT2R were designed by Shanghai Sangon Online, and are shown in Table 2. RT-qPCR amplification efficiencies were 90~110% for all primers and reagents. Briefly, the total reaction mixture (20 μL) consisted of SYBR Premix Ex Taq™ II (10 μL), the upstream and downstream primers (0.8 μL), ROX Reference Dye or Dye II (0.4 μL), cDNA (2 μL) and dH_2_O (6 μL). The following reaction conditions were used: 95 °C for 30 s, 95 °C for 5 s and 60 °C for 1 min for 40 cycles. β-actin was used as an internal reference gene, and data were analysed using the 2-^△△Ct^ method. The cell culture supernatant was collected, and Ang II and Ang-(1–7) levels were detected using ELISA kits according to the manufacturer’s instructions.

**Table 2:** **Sequences of the primers used for RT-qPCR**

Gene name	Accession number	Sequence of primers (5′ → 3′)	Product size (bp)
IL-1β	NM_174093.1	F: ATGAAGAGCTGCATCCAACACCTG R: ACCGACACCACCTGCCTGAAG	110
IL-6	NM_173923.2	F: CACTGACCTGCTGGAGAAGATGC R: CCGAATAGCTCTCAGGCTGAACTG	115
IL-8	AF232704	F: ATGACTTCCAAGCTGGCTGTTG R: TTGATA AATTTGGGGTGGAAAG	149
TNF-α	NM_173966.3	F: CTGGCGGAGGAGGTGCTCTC R: GGAGGAAGGAGAAGAGGCTGAGG	85
ACE2	XM_005228429	F: AGTGGTGGGAGATGAAGCGAGAG R: GGAACAGACACGCAGGATCACAG	87
ACE	NM_001206668.1	F: GGAGGTGCTGAAGGACATGGTTG R: CTCGCCGTTCTGCTGGTTCTG	112
MasR	XM_002690376	F: TGGGCTTTGTTGAGAACGGAATCC R: TGGGTGATGTAGACGGTGAAGGG	82
AT1R	XM_024990377	F: TCGCTTCAGCCAGTGTCAGTTTC R: ACAATAGCCAGGTAGCGGTCAATG	85
AT2R	XM_024988817	F: TGTATGGCTTGTCTGTCCTCATTGC R: GGCATACTTCTCAGGTGGGAAAGC	111
β-actin	NM_173979.3	F: CGTCCGTGACATCAAGGAGAAGC R: GGAACCGCTCATTGCCGATGG	143

### Western blot analysis

Total protein was extracted with a protein extraction kit (KGP2100, KeyGEN BioTECH, Jiangsu, China) according to the manufacturer’s protocol. Extracted proteins were quantified with BCA assays (ab102536, Abcam). Equal amounts of protein were separated by electrophoresis through 10 ~ 12% SDS-PAGE and transferred to PVDF membranes. The PVDF membrane was blocked in a 5% (m/v) skimmed milk for 2 h under gentle shaking. Then the dilution ratio of all primary antibodies was 1:1000 and incubated overnight at 4 °C. 1 × TBST was used to wash the membrane three times. HRP-labelled goat anti-rabbit IgG (ab6721 Abcam, 1:10 000) or goat anti-mouse IgG (ab205719, Abcam, 1:10 000) secondary antibody dilutions were incubated for 2 h at room temperature. 1 × TBST was used to wash the membrane three times, and this was followed by ECL chemiluminescence detection.

### Statistical analysis

All values are expressed as the mean ± standard error of the mean (mean ± SEM) of three independent experiments and were analysed by SPSS 22.0 software, and graphed by GraphPad Prism 8.0. Multiple samples were analysed using ANOVA, and the data among groups were compared in pairs using *Student–Newman–Keuls* (SNK) tests. The correlation of LPS, inflammatory factor, and components of the RAS was analysed using Spearman’s correlation analysis and imaged using R version 3.4.1. ns, represents no significant difference (*p* > 0.05); * represents *p* < 0.05; ** represents *p* < 0.01; and *** represents *p* < 0.001.

## Results

### Correlation analysis between LPS, inflammatory factors, and RAS pathway members

**Figure 1:**
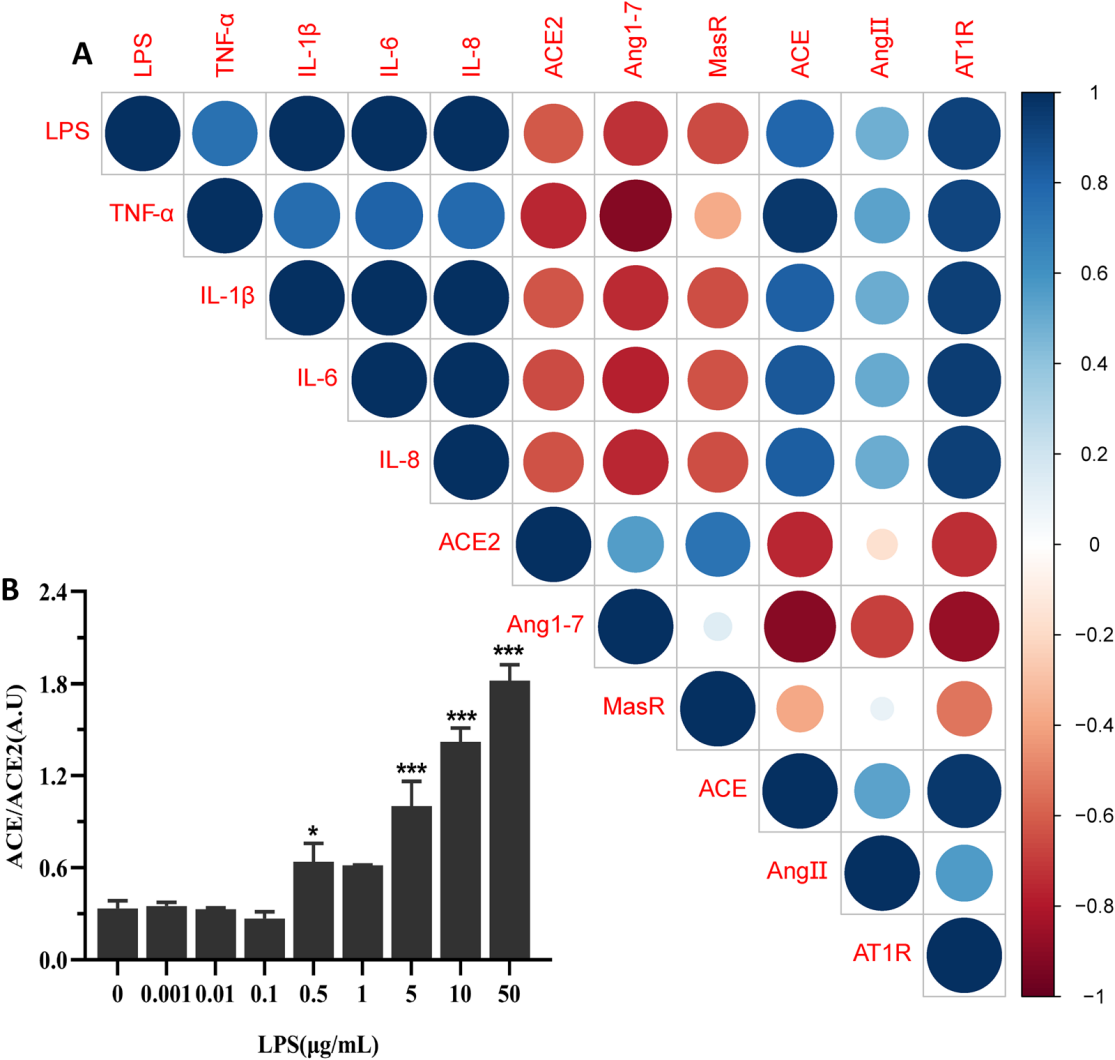
**Correlation analysis between LPS, inflammatory factors, and RAS pathway members. A** Heat map showing the correlation between LPS, inflammatory cytokines and RAS members. Blue dots represent positive correlation and red dots represent negative correlation. Larger dots and darker colour correspond to greater correlation. Lower correlation is represented by smaller dots and lighter colour. **B** Ratio of ACE/ACE2 after treatment of BMEC with different concentrations of LPS. All data were represented as the mean ± SEM (*n* = 3). ns, represents no significant difference (*p* > 0.05); * represents *p* < 0.05; ** represents *p* < 0.01; and *** represents *p* < 0.001.

The relationship between inflammatory cytokines and RAS pathway members during the LPS-induced inflammatory response in BMEC after treatment with different concentrations of LPS was examined by Spearman’s rank correlation coefficient analysis (Additional file 2, Additional file 3). TNF-α, IL-1β, IL-6, IL-8, ACE, Ang II and AT1R expression levels were significantly positively correlated with the concentration of LPS, while the expression of ACE2, Ang1-7 and MasR was significantly negatively correlated with the concentration of LPS (Figure 1A). These findings indicate that both axes of the RAS system are involved in LPS-induced inflammatory responses in BMEC. At low LPS concentrations, the ACE2/Ang1-7/MasR pathway is dominant, with high expression of ACE2, Ang1-7 and MasR, while at high LPS concentrations the ACE/Ang II/AT1R pathway is dominant, and ACE, Ang II and AT1R expression is increased.

The ratio of ACE mRNA/ACE2 mRNA was examined in the ACE2/Ang-(1–7)/MasR and ACE/Ang II/AT1R pathways after cells were treated with different concentrations of LPS. We found the ratio declines to the basal level seen in non-challenged controls at LPS concentrations of less than or equal to 0.1 μg/mL, suggesting that ACE2 expression was dominant, the ACE2/Ang-(1–7)/MasR pathway had been activated, and an inflammatory response had not been induced in the cells (Figure 1B). In contrast, at LPS concentrations greater than 0.1 μg/mL, the ACE/ACE2 ratio increased, indicating that ACE expression was dominant, the ACE/Ang II/AT1R pathway had been activated, and cells were in a pro-inflammatory state.

### DA and DAD3 promote ACE2 expression

**Figure 2:**
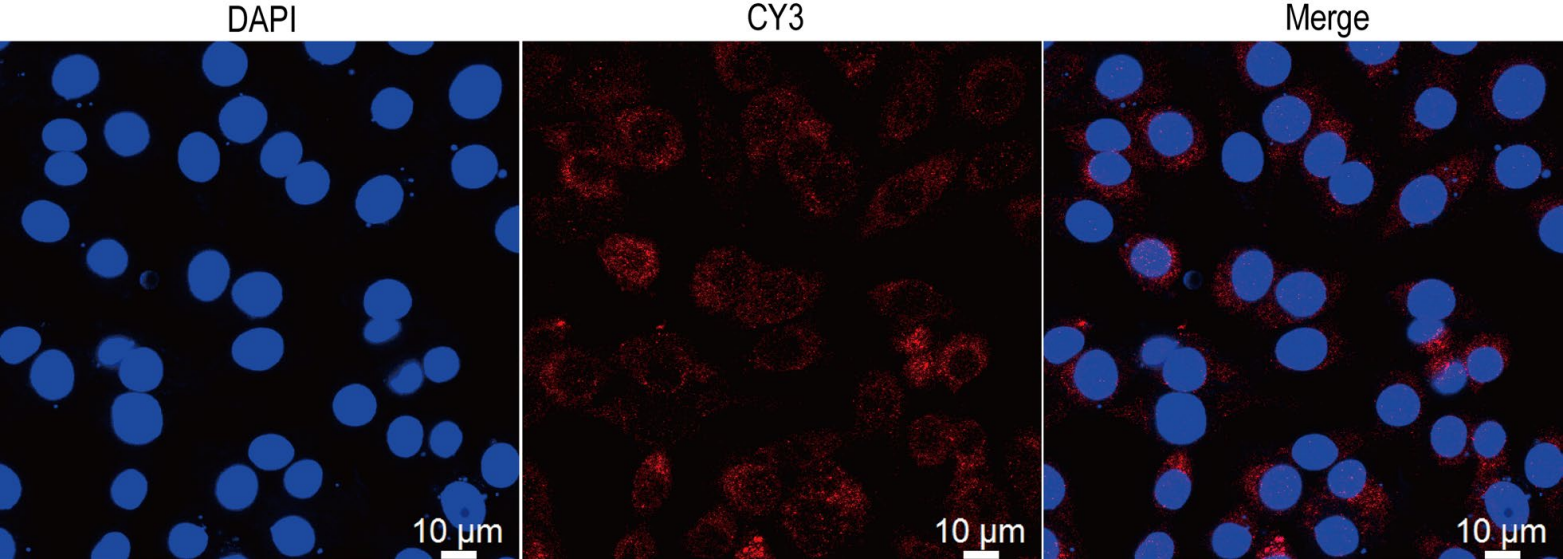
**Distribution of ACE2 in BMEC.** Cells were stained with antibodies against ACE2 and nuclei were stained with 4’,6-diamidino-2-phenylindole (DAPI). Representative images were visualized by confocal laser microscopy. Red, ACE2; blue, DAPI. Scale bar: 10 μm.

**Figure 3:**
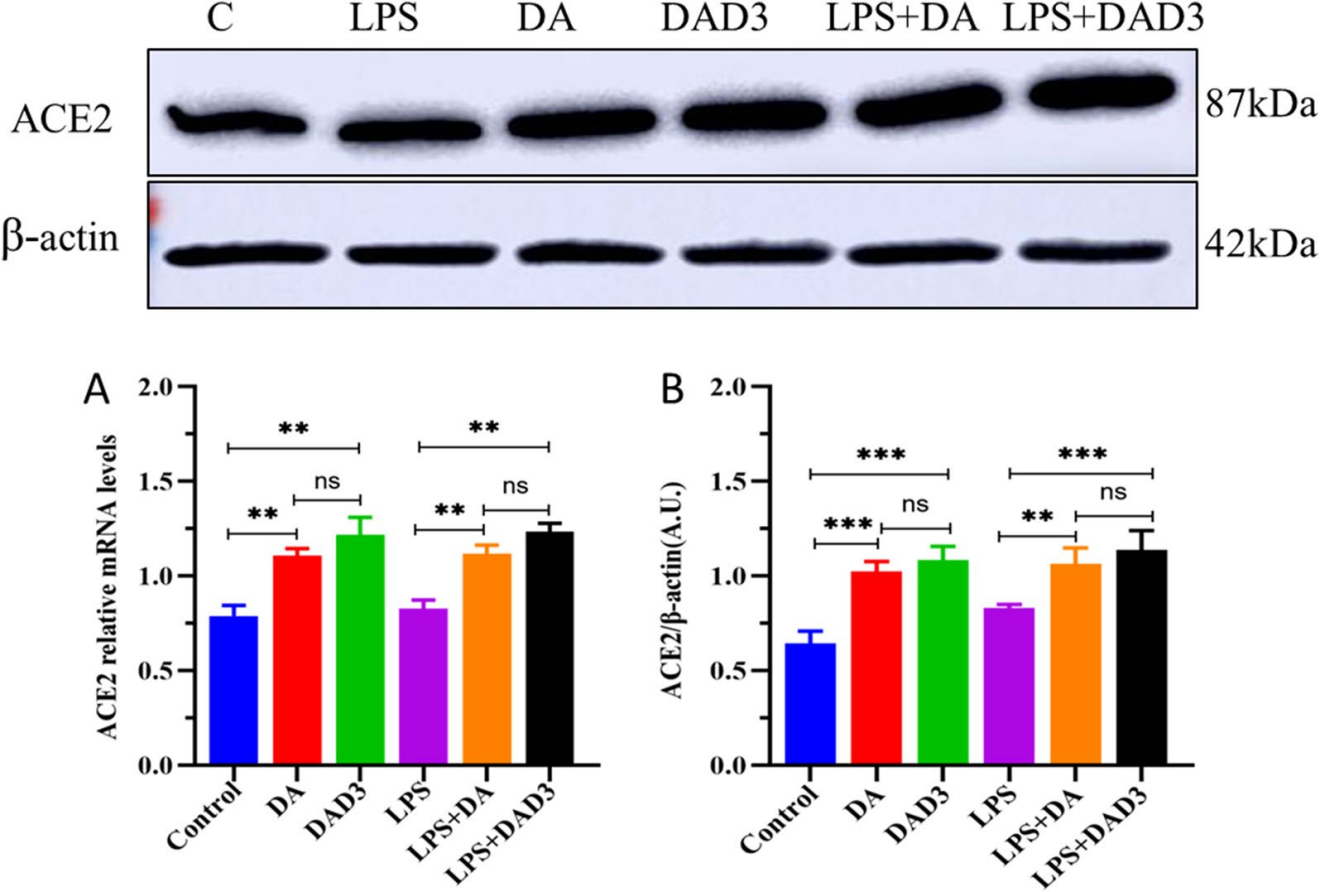
**DAD3 induces ACE2 expression in LPS-induced BMEC.** BMEC were treated with LPS (0.5 μg/mL), DA (20 μg/mL), DAD3 (20 μg/mL), LPS+DA (0.5 μg/mL+20 μg/mL) and LPS+DAD3 (0.5 μg/mL+20 μg/mL) for 24 h. **A** Relative ACE2 mRNA levels were determined by RT-qPCR. **B** ACE2 protein expression levels were determined by Western blotting. β-actin was used as a control. All data were represented as the mean ± SEM (*n* = 3). ns, represents no significant difference (*p* > 0.05); * represents *p* < 0.05; ** represents *p* < 0.01; and *** represents *p* < 0.001.

Laser confocal microscopy revealed that ACE2 protein was expressed in BMEC cytoplasm (Figure 2). Next, we examined the effects of DA and DAD3 on the transcription and expression levels of ACE2 in BMEC, and found a significant increase in ACE2 transcription and expression in the DA and DAD3 groups compared to the control group (Figures 3A and B). In addition, we found that the transcription and expression levels of ACE2 in the LPS+DA and LPS+DAD3 groups increased significantly compared to the LPS group (Figures 3A and B). These findings suggest that DA and DAD3 can activate and promote the transcription and expression of ACE2 in BMEC.

### ACE2 knockdown activates ACE/Ang II/ATIR pro-inflammatory axis and inhibits ACE2/Ang-(1–7)/MasR anti-inflammatory axis in DA- and DAD3-treated BMEC

**Figure 4:**
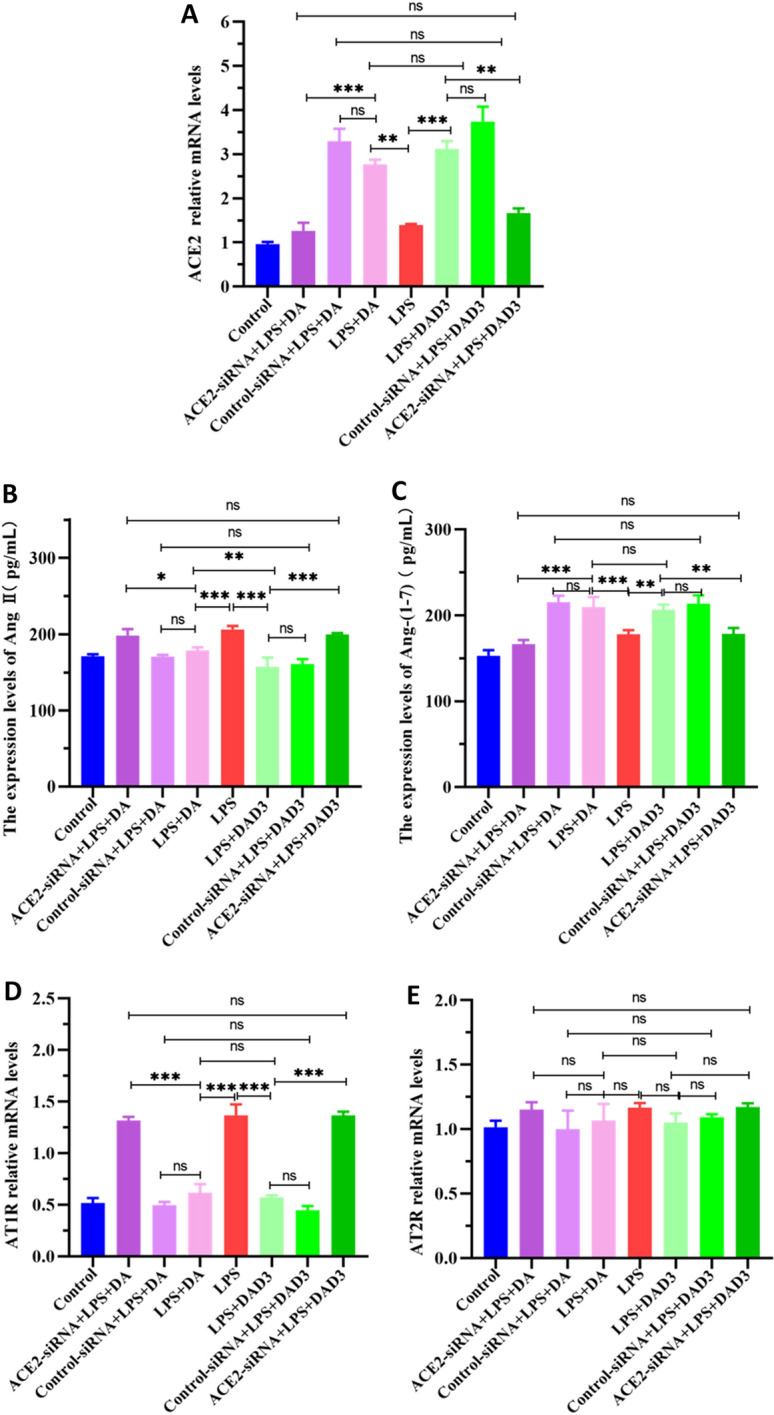
**Effects of ACE2 silencing and DAD3 treatments on the transcriptional or expression levels of RAS members. A-E** BMEC were treated with either control siRNA (scrambled) or ACE2-siRNA for 24 h, followed by treatment with 0.5 μg/mL LPS for 24 h. After washing with PBS, cells were treated with 20 μg/mL DA or 20 μg/mL DAD3 for 24 h. The ACE2, AT1R and AT2R relative mRNA levels (transcriptional levels) of RAS members were determined by RT-qPCR. The Ang II and Ang-(1–7) protein expression levels of RAS members were determined by ELISA. **A** Relative ACE2 mRNA levels. **B** Expression levels of Ang II. **C** Expression levels of Ang-(1–7). **D** Relative AT1R mRNA levels. **E** Relative AT2R mRNA levels. All data were represented as the mean ± SEM (*n* = 3). ns, represents no significant difference (*p* > 0.05); * represents *p* < 0.05; ** represents *p* < 0.01; and *** represents *p* < 0.001.

To determine whether the anti-inflammatory effects of DA and DAD3 are mediated through activation of ACE2, we treated ACE2-silenced BMEC with DA or DAD3 and examined the effects on RAS members. As shown in Additional file 4, ACE2-siRNA successfully silenced ACE2 expression. ACE2-siRNA-155 resulted in a more significant knockdown of ACE2 expression and was used for subsequent experiments. We found that ACE2 relative mRNA levels were significantly reduced in the ACE2-siRNA+LPS+DA and ACE2-siRNA+LPS+DAD3 treatment groups compared to the LPS+DA and the LPS+DAD3 groups, as expected after ACE2 knockdown by siRNA (Figure 4A). In addition, we found that Ang II expression was significantly reduced in the LPS+DA and LPS+DAD3 groups compared to the LPS group, while treatment with ACE2-siRNA led to a significant increase in Ang II expression (Figure 4B). In contrast, Ang-(1–7) expression levels in the LPS+DA and the LPS+DAD3 groups were significantly higher than the LPS group, and treatment with ACE2-siRNA led to a significant reduction in Ang-(1–7) expression levels (Figure 4C). AT1R relative mRNA levels were significantly increased after LPS treatment (LPS group), while treatment with DA or DAD3 (LPS+DA and LPS+DAD3 groups) significantly reduced AT1R transcription levels, and knockdown of ACE2 led to a significant increase in AT1R relative mRNA levels in DA and DAD3-treated cells (Figure 4D). However, AT2R relative mRNA levels were not affected by each treatment, and no significant differences were observed between the groups (Figure 4E). It is worth noting that no significant differences between DA or DAD3 (LPS+DA and LPS+DAD3 groups) and scrambled siRNA controls (siRNA+LPS+DA and siRNA+LPS+DAD3 groups) were observed for each RAS component, indicating that the scrambled siRNA controls used here were suitable controls for this study. These results indicated that ACE2 knockdown led to inhibition of DA and DAD3 agonistic effects, suggesting that these effects are mediated through ACE2. Furthermore, knockdown of ACE2 resulted in activation of the ACE/Ang II/AT1R axis, and inhibition of the ACE2/Ang-(1–7)/MasR axis.

### ACE2 knockdown inhibits DA and DAD3 anti-inflammatory effects

**Figure 5:**
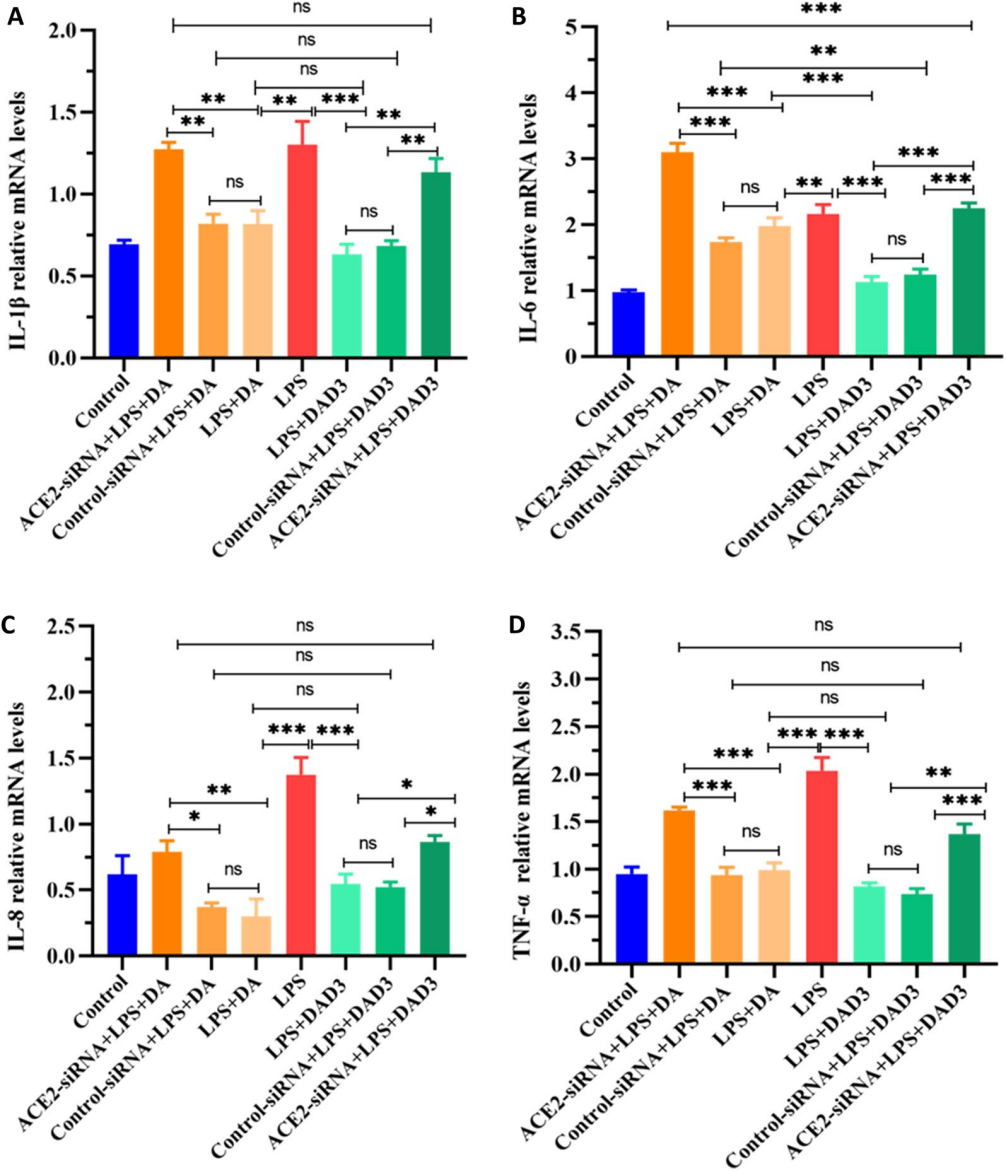
**Effects of ACE2 silencing and DAD3 treatments on the transcriptional levels of pro-inflammatory factors. A–D** BMEC were treated with either control siRNA (scrambled) or ACE2-siRNA for 24 h, followed by treatment with 0.5 μg/mL LPS for 24 h. After washing with PBS, cells were treated with 20 μg/mL DA or 20 μg/mL DAD3 for 24 h. Relative mRNA levels of pro-inflammatory factors were determined by RT-qPCR. **A** Relative IL-1β mRNA levels. **B** Relative IL-6 mRNA levels. **C** Relative IL-8 mRNA levels. **D** Relative TNF-α mRNA levels. All data were represented as the mean ± SEM (*n* = 3). ns, represents no significant difference (*p* > 0.05); * represents *p* < 0.05; ** represents *p* < 0.01; and *** represents *p* < 0.001.

In order to determine whether DA and DAD3 exert their anti-inflammatory effects through activation of ACE2, we used ACE2-siRNA to silence ACE2 expression and examined the relative mRNA levels of pro-inflammatory factors in LPS-induced BMEC treated with DA or DAD3. We found that the relative mRNA levels of IL-1β, IL-6, IL-8 and TNF-α were significantly increased in the ACE2-siRNA+LPS+DA group compared with the LPS+DA group (Figure 5). Similarly, IL-1β, IL-6, IL-8 and TNF-α relative mRNA levels were significantly increased in the ACE2-siRNA+LPS+DAD3 group compared with the LPS+DAD3 group (Figure 5). These results demonstrated that the anti-inflammatory effects of DA and DAD3 were inhibited after silencing ACE2 expression.

### ACE2 knockdown weakens DA and DAD3 inhibitory effects on the MAPK and NF-κB pathways

**Figure 6:**
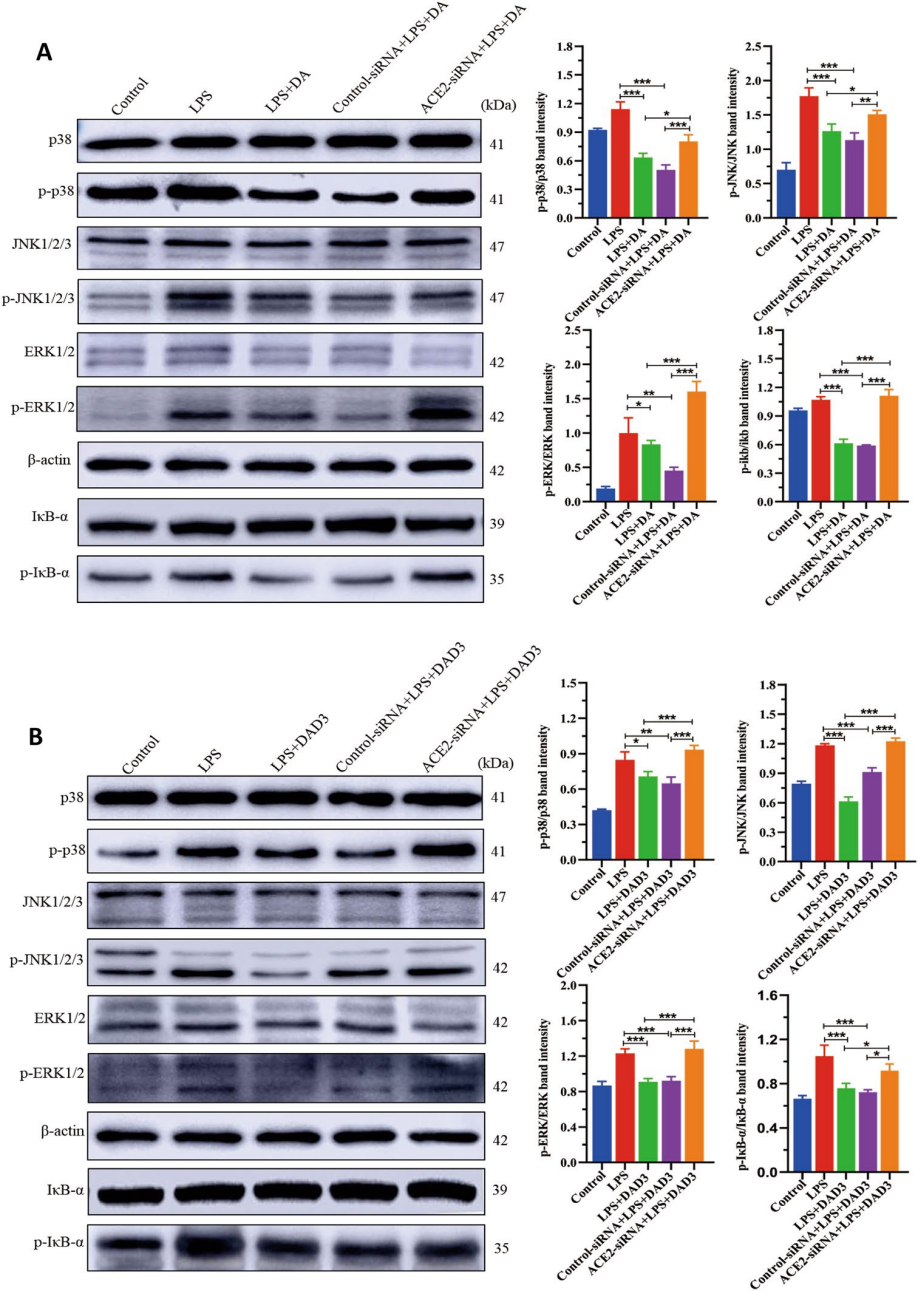
**Effects of ACE2 silencing and DA and DAD3 treatment on the expression levels of components of the MAPK and NF-κB pathways. A, B** BMEC were treated with either control siRNA (scrambled) or ACE2-siRNA for 24 h, followed by treatment with 0.5 μg/mL LPS for 24 h. After washing with PBS, cells were treated with 20 μg/mL DA or 20 μg/mL DAD3 for 24 h. The phosphorylation levels of p38, JNK1/2/3, ERK1/2 and IκB-α (p-p38, p-JNK, p-ERK1/2 and p-IκB-α) were determined by Western blotting. Activation of NF-κB pathway was indicated by p-IκB-α, an indicator for the activation of NF-κB. The band intensity of all detected proteins was normalized to β-actin and the expressions of p-p38, p-JNK, and p-ERK1/2 were normalized to p38, JNK, and ERK1/2, respectively. **A** DA treatments on the expression levels of components of the MAPK and NF-κB pathways; **B** DAD3 treatments on the expression levels of components of the MAPK and NF-κB pathways. All data were represented as the mean ± SEM (*n* = 3). ns, represents no significant difference (*p* > 0.05); * represents *p* < 0.05; ** represents *p* < 0.01; and *** represents *p* < 0.001.

In order to examine the mechanism by which DA and DAD3 inhibit production of inflammatory cytokines in LPS-induced BMEC, we assessed the expression levels of key MAPK pathway (p38, JNK1/2/3, and ERK1/2) and NF-κB pathway (IκB) proteins that regulate the production of pro-inflammatory cytokines. Western blot analysis revealed that compared with the control group, the phosphorylation levels of p38, ERK, JNK and IκB-α proteins in the LPS treatment group were significantly increased. Treatment of LPS-induced BMEC with DA, however, resulted in a significant decrease in the phosphorylation levels of p38, JNK, ERK and IκB-α compared to the LPS group (Figure 6A). Similarly, the phosphorylation levels of p38, JNK, ERK and IκB-α in the LPS+DAD3 group were significantly reduced compared to the LPS group (Figure 6B). These findings indicate that DA and DAD3 exert their anti-inflammatory effects by inhibiting the activation of MAPK and NF-κB pathways in LPS-stimulated BMEC.

Next, we examined the effects of ACE2 knockdown on the anti-inflammatory effects of DA and DAD3 in LPS-induced cells. We found that ACE2 knockdown (ACE2-siRNA+LPS+DA group) led to significant increases in the phosphorylation levels of p38, ERK and JNK1/2/3 proteins in the MAPK pathway, as well as IκB-α in the NF-κB pathway compared to the LPS+DA group (Figure 6A). Similar results were observed in the ACE2-siRNA+LPS+DAD3 group compared to the LPS+DAD3 group (Figure 6B). These results show that the inhibitory effects of DA and DAD3 on the MAPK and NF-κB pathways and their anti-inflammatory effects are dependent on ACE2. Thus, our findings suggest that the inhibitory effect of DA and DAD3 on LPS-induced inflammatory injury in BMEC is mediated through the activation of ACE2, which inhibits the phosphorylation of MAPK and NF-κB pathway proteins.

## Discussion

In the current study, we used Spearman’s correlation analysis to demonstrate that the ACE2/Ang-(1–7)/MasR and ACE/Ang II/AT1R pathways are dynamically balanced, having both positive and negative regulatory effects on each other. Furthermore, we showed that these two pathways are involved in the regulation of LPS-induced inflammatory injury in BMEC. At low LPS concentrations, we found that the ACE2/Ang-(1–7)/MasR pathway was dominant and exerted an anti-inflammatory effect, while at high LPS concentrations, the ACE/Ang II/AT1R pathway was found to be dominant and exerted a pro-inflammatory effect. Similarly, we showed that when the LPS concentration was less than or equal to 0.1 μg/mL, the ACE/ACE2 mRNA ratio decreased, indicating that ACE2 expression was dominant and that the ACE2/Ang-(1–7)/MasR pathway had been activated. In contrast, at LPS concentrations greater than 0.1 μg/mL, the ratio of ACE/ACE2 mRNA increased, indicating that ACE expression was dominant and that the ACE/Ang II/AT1R pathway had been activated. These findings confirmed the rationale of previous studies for treating BMEC with 0.5 μg/mL LPS to establish a cell inflammation model [22].

Previous studies have shown that in a pathological state, the ratio of ACE/ACE2 increases in the body, disrupting the RAS system, and resulting in increased injury [28, 29]. In the LPS-induced acute respiratory distress syndrome rat model, for example, the ratio of ACE/ACE2 was increased in the bronchoalveolar lavage fluid, resulting in activation of the ACE/AT1R/Ang II pathway, and increased expression of inflammatory factors [30]. Other studies have demonstrated that when inflammation occurs, the ratio of ACE2/ACE decreases, the ACE/AT1R/Ang II pathway is activated, and the expression of downstream proteins (Akt, ERK, eNOS) increases, leading to increased expression of pro-inflammatory factors (Profilin-1, MCP-1, IL-1β, IL-6) [31]. DA has a similar structure to the ACE2 activator XNT and has been shown to be an effective activator of ACE2 [13]. In this study, we found that ACE2 expression significantly increased in the DA and DAD3 groups compared with the control group. Similarly, ACE2 protein expression levels were significantly increased in the LPS+DA and LPS+DAD3 groups compared with the LPS group, indicating that DA and DAD3 can activate ACE2 and promote ACE2 expression. A previous study found that the ACE2/ACE ratio decreased in LPS-induced pulmonary microvascular endothelial cells, and that when the ACE2/ACE ratio increased, the ACE2/Ang-(1–7)/MasR pathway was activated, with increased Ang1-7 expression and decreased transcription of IL-1β and TNF-α. However, these protective effects were reduced when ACE2 expression was silenced or cells were treated with the selective MasR antagonist, A779 [13]. In this study, we found that when BMEC ACE2 was silenced by siRNA, Ang II and AT1R expression levels were significantly increased in the ACE/Ang II/AT1R axis in the ACE2-siRNA+LPS+DA and ACE2-siRNA+LPS+DAD3 groups, indicating that when ACE2 expression was silenced, the ACE/Ang II/ATIR axis was activated, and inflammation was observed in the cells. Correspondingly, Ang1-7 expression levels were significantly reduced in the ACE2-siRNA+LPS+DA and ACE2-siRNA+LPS+DAD3 treatment groups, indicating that the ACE2/Ang-(1–7)/MasR axis was inhibited, and BMEC were in a pro-inflammatory state. These findings demonstrate that the inhibitory effects of DA and DAD3 on BMEC inflammation are ACE2-dependent, suggesting that ACE2 may be the main target of DA and DAD3. Previous studies have shown that DA had no significant effect on the expression of AT2R, which is consistent with our findings [18]. This may be because AT2R expression in BMEC is not affected by LPS stimulation, or because LPS-induced Ang II binds to AT1R, and not AT2R.

DA can alleviate the endotoxin-induced inflammatory response and inflammatory cell infiltration in the mouse eye by activating the ACE2/Ang-(1–7)/MasR axis [32, 33]. Here, we show that DA and DAD3 can promote ACE2 expression to inhibit the expression of TNF-α, IL-1β, IL-6 and IL-8 in LPS-induced BMEC. However, when ACE2 is silenced, the inhibitory effects of DA and DAD3 are reduced. Studies have shown that the ACE2/Ang-(1–7)/MasR axis can inhibit inflammation by inhibiting the MAPK and NF-κB signalling pathways in an in vitro pancreatitis model [34]. Similarly, AAV gene transfer has been shown to activate the ocular ACE2/Ang-(1–7)/MasR axis and inhibit the MAPK, NF-κB and STAT3 signalling pathways to prevent experimental autoimmune uveitis [35]. Furthermore, in the LPS-induced inflammation model of omental pigment epithelial cells, DA significantly down-regulated IL-6, IL-8 and MCP-1 expression, while up-regulating ACE2 expression, resulting in inhibition of p38, ERK1/2, JNK and p-IκB phosphorylation. However, these protective effects of DA are reduced when ACE2 expression is silenced or cells are treated with A779 [18]. Our study confirmed that DA and DAD3 treatment reduced production of the inflammatory cytokines TNF-α, IL-1β, IL-6 and IL-8, inhibited Ang II and AT1R expression, upregulated Ang1-7 and MasR expression, and inhibited p38, JNK, ERK and IκB-α protein phosphorylation in BMEC. However, these protective effects were abolished after ACE2 expression was silenced. Thus, our findings indicate that like DA, DAD3 targets ACE2 to inhibit the MAPK and NF-κB signalling pathways and protect against LPS-induced BMEC inflammation.

In summary, we demonstrate that anti-pro-inflammatory cytokines may be a potential therapeutic strategy in the treatment of mastitis. Furthermore, our results provide useful and additional mechanisms for the anti-inflammatory effects of DAD3. Our findings indicate that DAD3 may be a potential therapeutic agent against mastitis. However, these results are based on studies at the cellular level, and thus, further studies in vivo are required to confirm the potential use of DAD3 in the treatment of mastitis.

DA and DAD3 have an agonistic effect on ACE2 in BMEC. DA and DAD3 can regulate the balance of the ACE/Ang II/AT1R and ACE2/Ang-(1–7)/MasR axes in BMEC by activating ACE2. DA and DAD3 inhibit LPS-induced inflammation of BMEC by activating ACE2 and inhibiting the MAPK and NF-κB signalling pathways.

## Supplementary Information

**Additional file 1: Synthetic route of 2-methylbenzenesulfonyl chloride and DA complexes.** DAD3:4,4´ -(1-Triazene-1,3-) bisbenzyl2-methylbenzenesulfonamide hydrochloride.

**Additional file 2: Expression levels of RAS members in BMEC treated with different concentrations of LPS.**

**Additional file 3: Expression levels of pro-inflammatory factors in BMEC treated with different concentrations of LPS.**

**Additional file 4: ACE2-siRNA silences ACE2 expression in BMEC**. ACE2 protein expression levels were detected in cells treated with ACE2-siRNA-155 and ACE2-siRNA-2321 by Western blotting. The expression level of ACE2 protein in the ACE2-siRNA-155 and ACE2-siRNA-2321 interference groups was significantly decreased (*P* < 0.01) compared with the negative control group (NC group), which indicated that the two pairs of siRNA could inhibit the expression of ACE2 protein. In addition, ACE2-siRNA-155 resulted in significantly reduced expression of ACE2 protein compared to ACE2-siRNA-2321 (*P* < 0.01), indicating that the interference effect of ACE2-siRNA-155 was better. Based on these findings, ACE2-siRNA-155 was used in subsequent experiments. BC group: Blank control group; NC group: negative control group; ACE2-siRNA-155 and ACE2-siRNA-2321: interference group. All data were represented as the mean ± SEM (*n* = 3). ns, represents no significant difference (*p* > 0.05); * represents *p *< 0.05; ** represents *p *< 0.01; and *** represents *p *< 0.001.

## Data Availability

All data generated or analysed during this study are included in this published article.

## Abbreviations

RAS: renin-angiotensinsystem
ACE: angiotensin-converting enzyme
ACE2: angiotensin-converting enzyme 2
Ang II: angiotensin II
Ang-(1–7): angiotensin 1–7
AT1R: angiotensin II Type 1 receptor
AT2R: angiotensin II Type 2 receptor
MAPK: mitogen-activated protein kinase
NF-κB: nuclear factor kappa-B
