# Supplementary figures and images for: DAD3 targets ACE2 to inhibit the MAPK and NF-κB signalling pathways and protect against LPS-induced inflammation in bovine mammary epithelial cells
Source: Vet Res. 2022 Dec 8;53:104. doi: 10.1186/s13567-022-01122-0 (PMC9733329; doi:10.1186/s13567-022-01122-0)

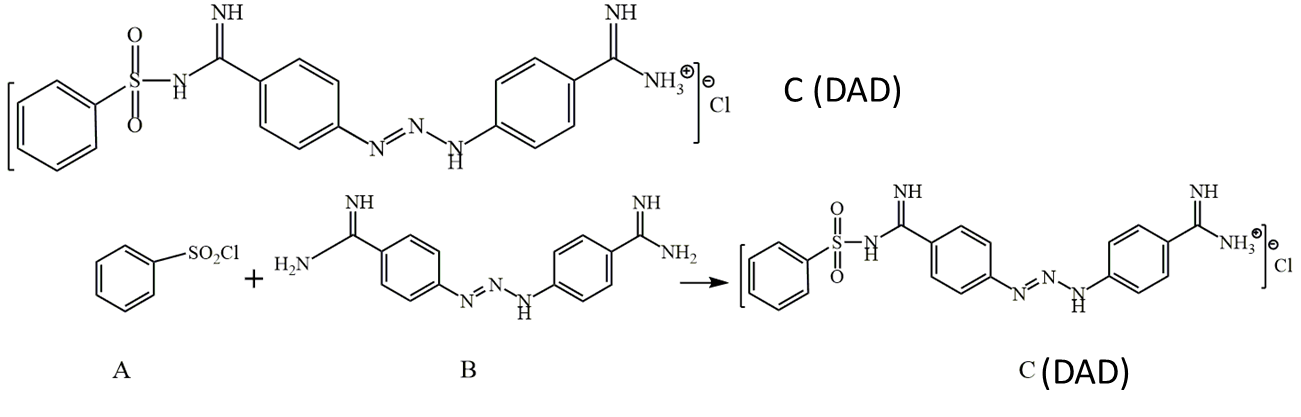

Supplement: Supplementary file 1 — Additional file 1: Synthetic route of 2-methylbenzenesulfonyl chloride and DA complexes. DAD3:4,4´ -(1-Triazene-1,3-) bisbenzyl2-methylbenzenesulfonamide hydrochloride. [file 13567_2022_1122_MOESM1_ESM.tif]

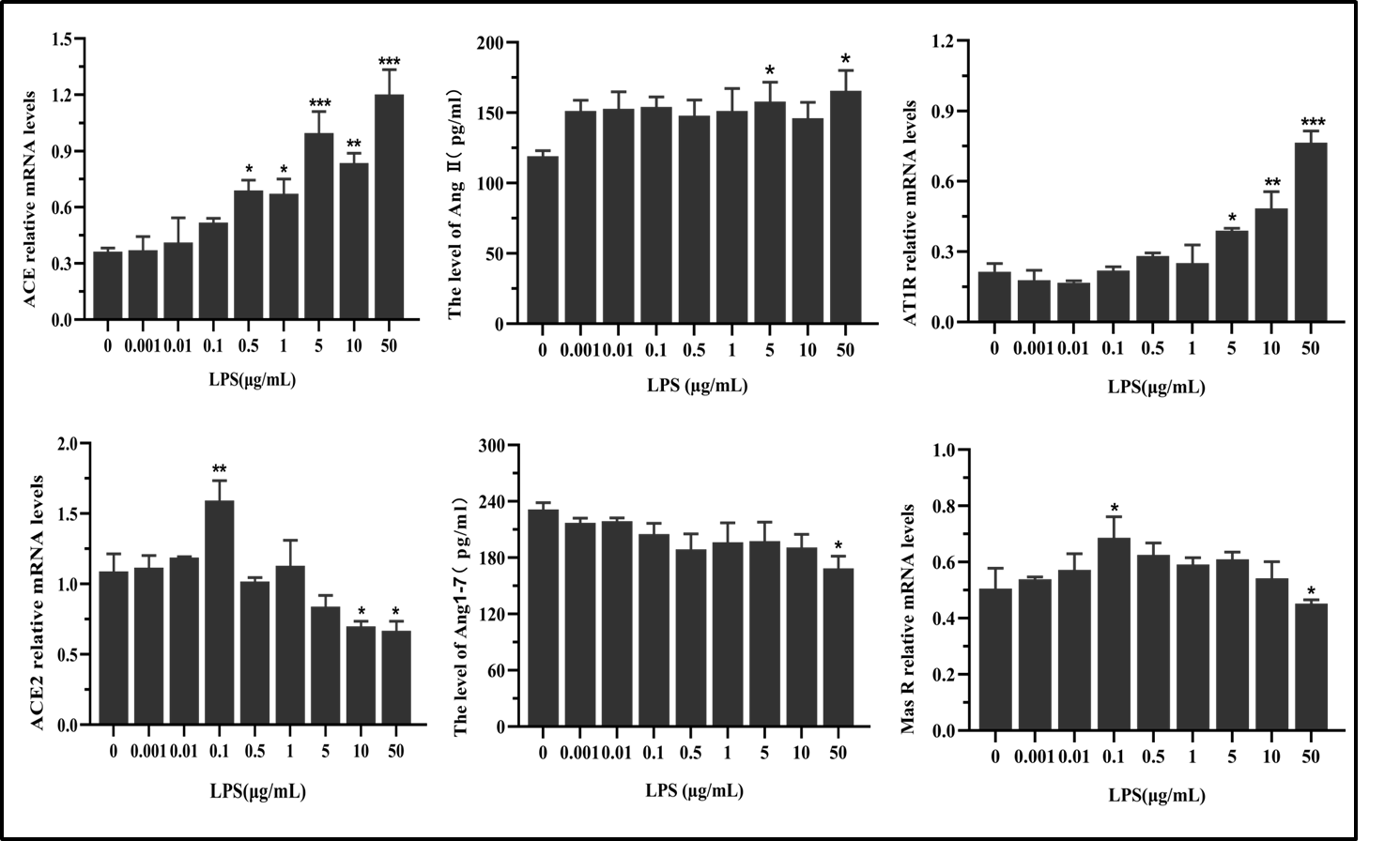

Supplement: Supplementary file 2 — Additional file 2: Expression levels of RAS members in BMEC treated with different concentrations of LPS. [file 13567_2022_1122_MOESM2_ESM.tif]

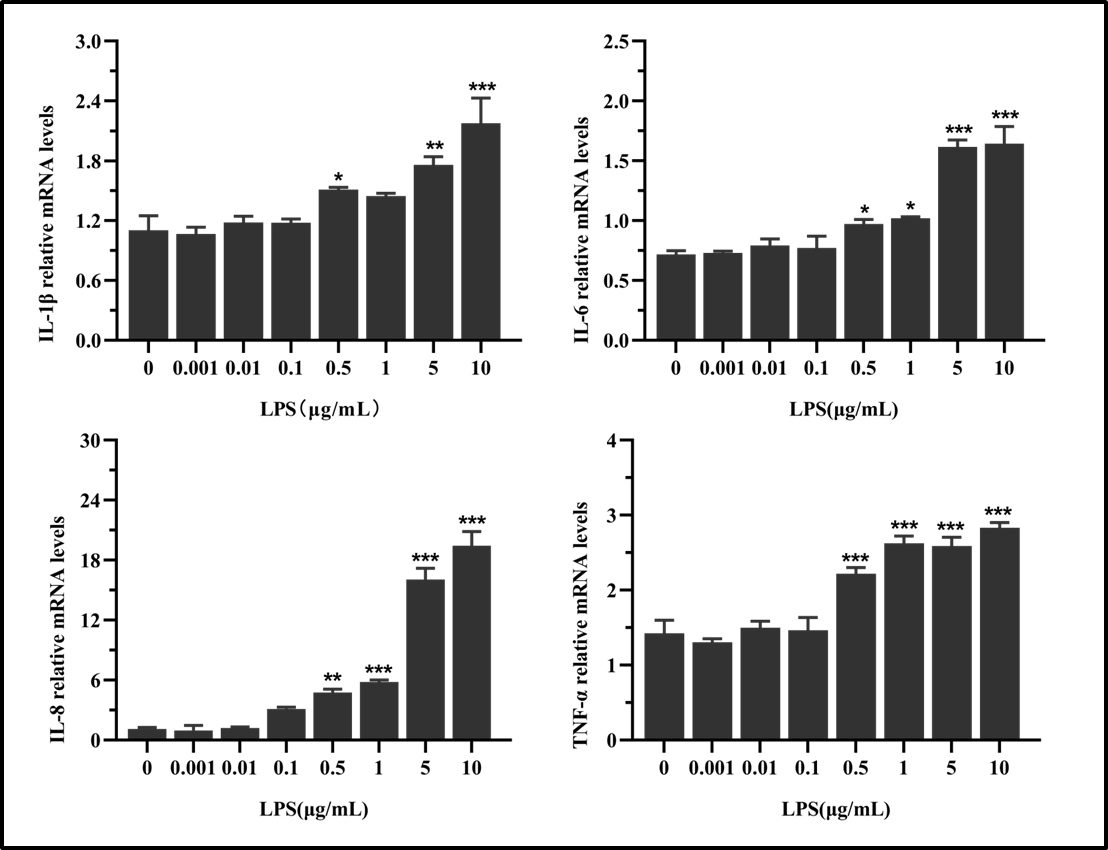

Supplement: Supplementary file 3 — Additional file 3: Expression levels of pro-inflammatory factors in BMEC treated with different concentrations of LPS. [file 13567_2022_1122_MOESM3_ESM.tif]

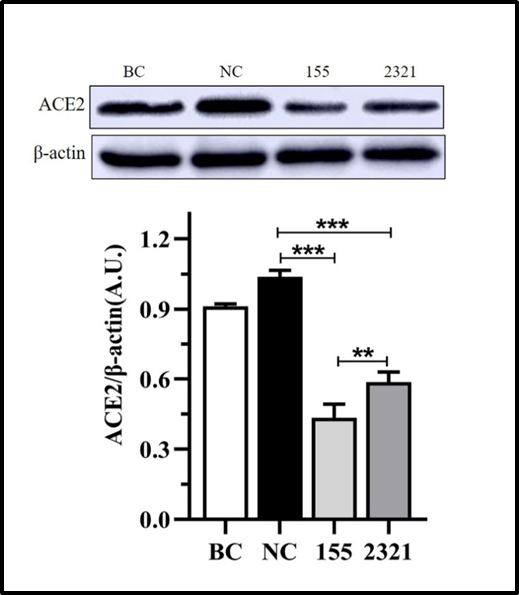

Supplement: Supplementary file 4 — Additional file 4: ACE2-siRNA silences ACE2 expression in BMEC. ACE2 protein expression levels were detected in cells treated with ACE2-siRNA-155 and ACE2-siRNA-2321 by Western blotting. The expression level of ACE2 protein in the ACE2-siRNA-155 and ACE2-siRNA-2321 interference groups was significantly decreased (P < 0.01) compared with the negative control group (NC group), which indicated that the two pairs of siRNA could inhibit the expression of ACE2 protein. In addition, ACE2-siRNA-155 resulted in significantly reduced expression of ACE2 protein compared to ACE2-siRNA-2321 (P < 0.01), indicating that the interference effect of ACE2-siRNA-155 was better. Based on these findings, ACE2-siRNA-155 was used in subsequent experiments. BC group: Blank control group; NC group: negative control group; ACE2-siRNA-155 and ACE2-siRNA-2321: interference group. All data were represented as the mean ± SEM (n = 3). ns, represents no significant difference (p > 0.05); * represents p < 0.05; ** represents p < 0.01; and *** represents p < 0.001. [file 13567_2022_1122_MOESM4_ESM.tif]
